# Experiences of severe burn survivors in the hospital-to-home transition

**DOI:** 10.1590/0034-7167-2024-0493

**Published:** 2025-10-03

**Authors:** Karina da Silva Vale Yagi, Vanessa da Silva Carvalho Vila, Maysa Ferreira Martins Ribeiro, Virginia Visconde Brasil

**Affiliations:** I Pontifícia Universidade Católica de Goiás. Goiânia, Goiás, Brazil; II Universidade Federal de Goiás. Goiânia, Goiás, Brazil

**Keywords:** Burns, Patient Discharge, Continuity of Patient Care, Nursing Care, Transitional Care., Quemaduras, Alta del Paciente, Continuidad de la Atención al Paciente, Atención de Enfermería, Cuidado de Transición.

## Abstract

**Objective:**

To understand the lived experience of severe burn survivors during the hospital-to-home transition.

**Methods:**

This qualitative study was conducted with 13 survivors (eight women and five men) at a burn reference center in Goiânia, Brazil. Data collection included medical records, phone contacts to confirm participation, and both remote and in-person interviews. The interviews were recorded, transcribed, and analyzed through interpretative thematic analysis in six stages.

**Results:**

The main challenges faced by participants were physical sequelae (pain, scars, and changes in body image) and emotional distress. Barriers to returning to daily activities included social exclusion, job displacement, reduced income, and difficulty accepting the new body image, while family support was essential.

**Conclusion:**

The hospital-to-home transition requires comprehensive and continuous care, with a focus on social and occupational reintegration, as well as emotional support.

## RESUMEN

**Objetivo:**

Comprender la experiencia de sobrevivientes de quemaduras graves en el proceso de transición del hospital al domicilio.

**Métodos:**

Estudio cualitativo realizado con 13 sobrevivientes (ocho mujeres y cinco hombres) en un centro de referencia en Goiânia, Brasil. La recolección de datos incluyó revisión de expedientes clínicos, contactos telefónicos para confirmar la participación y entrevistas presenciales y remotas. Las entrevistas fueron grabadas, transcritas y analizadas mediante análisis temático interpretativo en seis etapas.

**Resultados:**

Los principales desafíos fueron las secuelas físicas (dolor, cicatrices, alteraciones en la imagen corporal) y el sufrimiento emocional. Entre las barreras para el retorno a la vida cotidiana se destacaron la exclusión social, el alejamiento laboral, la disminución de ingresos y las dificultades para aceptar la nueva imagen corporal, mientras que el apoyo familiar fue esencial.

**Conclusión:**

La transición poshospitalaria requiere cuidados integrales y continuos, con enfoque en la reinserción social, laboral y el soporte emocional.

## INTRODUCTION

Burn injuries are tissue injuries caused by a variety of agents, including chemical, thermal, physical, and biological elements, resulting in traumatic wounds with significant physical, psychological, and aesthetic implications^(1,2)^. Individuals who survive burn injuries face multifaceted challenges that extend beyond the risks associated with the loss of tissue integrity and include the management of severe pain, emotional distress-including anxiety and stress-and difficulties adapting to the physical and functional changes imposed by the injury^(1-3)^.

Globally, burn injuries are recognized as a serious public health issue, with approximately 180,000 deaths recorded annually, while in Brazil, about 2,500 deaths each year are attributed to this cause^(1)^. The incidence of these injuries is more pronounced in lowand middle-income countries and is influenced by unfavorable socioeconomic conditions, such as precarious housing, lack of education, overcrowded living environments, and deficiencies in health education and prevention campaigns^(4)^.

Nonfatal burn injuries often result in long-term complications, including physical limitations, permanent scarring, and psychological trauma, all of which impair quality of life and individual autonomy, affecting daily activities, social interactions, and overall well-being^(1,5)^. The severity of the injury is determined by the extent of the affected total body surface area (TBSA) and the depth of the burn, with severe injuries involving more extensive tissue damage and a higher risk of mortality^(6)^.

Following hospitalization, the role of the family becomes crucial in the patient’s recovery process by providing emotional support and assisting with treatment adherence^(7)^. The hospital-to-home transition requires a multidisciplinary approach to identify the individual needs of both the patient and their family^(8)^. However, hospital discharge planning is often overlooked, resulting in inadequate transitions that can compromise patient recovery and hinder community reintegration^(9,10)^.

Understanding the lived experiences of burn survivors can offer valuable insights for developing interventions and policies to enhance the support provided during the hospital-to-home transition, addressing a significant gap in both the literature and clinical practice.

## OBJECTIVE

To understand the lived experience of severe burn survivors during the hospital-to-home transition.

## METHODS

### Ethical aspects

The research ethics committees of the Pontifical Catholic University of Goiás and the Leide das Neves Ferreira Center for Assistance to Radiation Accident Victims approved the study, which complied with the ethical principles established in Resolution 466/2012 of the Brazilian National Health Council. The Informed Consent Form was obtained from all study participants, either in printed or digital format, according to each participant’s preference. Survivors were identified by the letter P followed by an Arabic numeral.

### Study design, period, and setting

This is a qualitative study reported in accordance with the Consolidated Criteria for Reporting Qualitative Research (COREQ)^(11)^. Data was collected between January and June 2022 at a High Complexity Burn Care Reference Center in the metropolitan region of Goiânia, state of Goiás, Brazil.

### Sample, inclusion and exclusion criteria

The study included survivors of severe burn injuries who were receiving follow-up care at the burn reference center during the data collection period. Participant selection began with identifying individuals who had been discharged from the hospital between 30 and 180 days prior to data collection, ensuring they had undergone an initial period of home adaptation, which was essential for capturing their experiences.

The inclusion criteria were: age 18 years or older; severe and complex burn injuries, defined as burns involving more than 20% of the total body surface area (TBSA), deep second-degree burns or greater, burns in critical areas, and/or electrical burns; hospital discharge between 30 and 180 days prior to data collection; and follow-up care by the plastic surgery team.

Patients followed by the plastic surgery team for reasons other than burn injuries, individuals whose hospital discharge had occurred more than six months before data collection, and those who had discontinued outpatient treatment were excluded. The assessment of burn severity was performed alongside verification of the inclusion criteria, as the study adopted specific parameters for this classification. Thus, the inclusion criteria already encompassed the definition of severity used.

During data collection, 150 potential participants were identified based on the list of patients scheduled for follow-up visits with the burn center’s multidisciplinary team. By applying the inclusion criteria, 50 individuals were found to be eligible for the study and were contacted. However, 37 were lost to follow-up due to outdated or invalid phone numbers and participation refusals. Therefore, the final sample comprised 13 survivors of severe burn injuries.

Since participant selection was based on the availability of patients receiving follow-up care at the burn reference center and on the feasibility of contacting and recruiting them, the sample was characterized as a non-probability convenience sampling. This sampling method was adopted considering the specificities of the study, including the need to identify survivors of severe burn injuries within a specific hospital discharge timeframe and receiving outpatient care, as well as the feasibility of recruitment within the established research period.

### Data collection

Data collection was conducted in two stages. Initially, participants were identified and selected based on clinical records available in the institution’s electronic medical record system. This process enabled the analysis of burn severity criteria and the application of the study’s inclusion and exclusion criteria. The researcher obtained authorization to access the list of patients scheduled for follow-up visits with the plastic surgery team. This allowed for the review of medical reports, nursing records, and notes from the multidisciplinary team, covering the period from initial care through hospital discharge.

After this initial stage, a list containing basic patient information, such as phone contacts and a brief hospitalization history, was organized. During phone contacts, it was found that most of the registered numbers did not belong directly to the survivors but to family members, friends, or support institutions. For this reason, two to three call attempts were necessary to establish the first direct contact with the participant. During the calls, the researcher identified herself, explained the study’s objectives, and provided all necessary information so that survivors could make an informed decision about participating. After verbal confirmation of interest, participants were offered the option of either an online or in-person interview.

Two in-person interviews and eleven online interviews were conducted, guided by a semi-structured interview script exploring sociodemographic aspects and experiences related to living with a burn injury. Examples of questions included: “Did you face any difficulties when returning home?”, “How do you feel today about having experienced a burn injury?”, and “What helped you cope with this process?” All interviews were recorded and numerically coded to preserve participant anonymity.

Data collection was concluded through an analytical, interactive, and interpretive process, following the principle of theoretical sufficiency, which considers the quality and depth of the information obtained as key factors in ending data collection. The decision to conclude data collection was based on analytical coherence, ensuring that emerging themes were sufficiently developed; diversity and variety, ensuring multiple perspectives on the post-burn experience; conceptual density, with themes consistently supported by detailed descriptions; and a pragmatic criterion, considering time feasibility and participant availability.

This approach enabled a careful closure of the data collection process, allowing thematic analysis to be conducted rigorously and aligned with the methodological principles of the reflexive approach. Thus, we emphasized that the decision to conclude data collection was not based solely on the absence of new information but rather on the sufficiency of the information to address the study’s objectives, providing interpretive depth and coherence.

### Data analysis

Initially, the interviews were transcribed shortly after each session with the assistance of one of the study’s authors, following training, guidance, and supervision by the principal investigator. The data were then analyzed using Reflexive Thematic Analysis^(12)^, an interpretive method that emphasizes the researcher’s active construction of meaning, allowing for an in-depth and theoretically informed reading of the phenomenon under study. The approach adopted was inductive, meaning themes emerged directly from the data without imposing predefined categories, enabling the survivors’ experiences to be analyzed in their complexity and uniqueness.

The analytical process followed six interconnected stages. First, familiarization with the data was undertaken, involving a careful reading of the transcripts and initial notes on emerging patterns. Next, coding was carried out through line-by-line analysis to highlight relevant units of meaning. Subsequently, codes were grouped into themes, organizing them into broader patterns of meaning based on their interrelationships. This structure was refined during the theme review stage, promoting internal coherence and clear distinctions between themes. Following this refinement, the themes were defined and named, consolidating their central meanings and articulating their theoretical foundations. Finally, the themes were described and interpreted narratively, considering the depth and complexity of the meanings expressed by the survivors.

As the material was read in greater depth, codes were identified progressively across all interviews, using an iterative and reflexive approach. This step included a critical data reading, analytical discussions with the principal investigator, and a continuous review of emerging codes. The construction of themes occurred through the progressive grouping and refinement of codes, culminating in structuring macrothemes that synthesized participants’ experiences.

The decision to conclude the analysis followed the theoretical sufficiency principle, prioritizing the themes’ depth and interpretive coherence rather than the traditional notion of data saturation. This approach emphasizes a continuous and reflexive analytical process in which the quality of interpretation prevails over the mere repetition of information. A set of criteria was adopted to guide the definition of the analysis endpoint, ensuring methodological clarity^(12)^.

The first criterion was conceptual density, considering that the emerging themes demonstrated consistency and depth, supported by participants’ detailed descriptions, without the need for additional data to enhance the understanding of the phenomenon. Next, internal coherence was evaluated to ensure that the identified codes and themes maintained logical and well-defined relationships, reflecting meaningful patterns within the dataset.

The variability of experiences was also taken into account to ensure that different perspectives were represented in the analysis without requiring a greater volume of interviews to reinforce already established patterns. The analytical stability criterion was likewise considered, verifying that data interpretation remained consistent throughout the different stages of analysis without new conceptual variations emerging.

Another fundamental aspect was peer debriefing, carried out with the principal investigator and other research team members, which allowed for a critical and collaborative review of the findings and strengthened the interpretive validity of the analysis. Finally, pragmatic aspects were considered, such as the study’s time feasibility and participant availability, to balance theoretical depth and study feasibility. Based on these criteria, the analysis was conducted carefully and iteratively to ensure fidelity to the experiences narrated by the participants and internal coherence of the identified themes.

## RESULTS

The study included 13 burn survivors, eight (61.5%) of whom were women and five (38.5%) men, aged between 22 and 42 years (mean age: 34.2 years). Six participants (46.2%) were married or lived in a common-law marriage, five (38.5%) were never married, and two (15.3%) were divorced. Nine participants (69.2%) identified as Protestant and four (30.8%) as Catholic. Eight survivors (61.5%) had between one and three children. Nine participants (69.2%) had completed high school. The majority reported a household income between one and two times the minimum wage. Regarding occupation, six (46.2%) had formal employment, one (7.7%) was on temporary disability leave, and five (38.5%) were self-employed without a fixed income.

Data analysis revealed that the hospital-to-home transition was a challenging process for survivors of severe burn injuries, involving multiple dimensions that influenced their adaptation to a new reality. Six macrothemes emerged from the inductive analysis, representing the main experiences and challenges faced by the survivors: Difficulties returning to daily life; Impact on self-image and social life; Financial challenges and impact on the role of provider; Difficulties accessing treatment and maintaining continuity of care; Overcoming challenges and receiving support in rebuilding life; and Faith and spirituality.

These macrothemes structure the presentation of the results and provide a comprehensive understanding of the survivors’ experiences. The findings are illustrated with excerpts from the survivors’ narratives, highlighting the nuances and meanings attributed to each aspect experienced during the post-discharge period.

### Difficulties returning to daily life

Returning home was described by survivors as a moment of intense insecurity and fear. The absence of the hospital team created a sense of helplessness, making adaptation even more challenging.

*It’s scary!* [...] *I’m still kind of* [...] *in disbelief!* [...] *I keep thinking* [...] *am I really the one going through this?!* (P2)

*Coming back is very painful, everything makes you suffer.* (P5)

Persistent pain was also reported as one of the main challenges, often uncontrollable with the prescribed medications. Some survivors sought relief on their own without professional guidance.

*Everything you do, you feel pain* [...] *it’s a lot of pain, an indescribable pain!* [...] *my skin is extremely sensitive* [...] *you feel stabbing pains, you never forget. Even when you try to, the wound itself reminds you that it’s there!* (P9)

*The doctor who discharged me didn’t prescribe any pain medication, only dipyrone!* [...] *I spent about three days at home in pain after I got back!* [...] *One day I was crying a lot, I couldn’t stand it! I called my neighbor, who’s a firefighter nurse!* [...] *she managed to get me a prescription for tramadol.* (P1)

Motor limitations and the need to avoid sun exposure also impacted survivors’ autonomy by restricting mobility and increasing dependence on others for daily activities.

*I’m very limited* [...] *I can’t go out in the sun* [...] *I still can’t play volleyball* [...] *I can’t run* [...] *I can’t even walk properly.* (P1)

### Impact on self-image and social life

Acceptance of the new body image was one of the greatest challenges faced by survivors. Scarring and hair loss were sources of distress and low self-esteem, making it difficult for them to recognize their own appearance.

*I think it takes a while to get used to all of this!* [...] *I haven’t accepted what happened!* [...] *I feel rejection toward myself, toward looking in the mirror* [...] *The self-esteem I had before, I don’t have anymore!* (P8)

*I try not to look in the mirror; the scars are deep and horrible! If I look, I get sad!* [...] *If I could have corrective surgery, I would. I’m not afraid!* (P12)

The perception of others’ gazes was also an impactful factor. Many survivors reported discomfort and embarrassment in public spaces, feeling observed and, at times, excluded from social interactions.

*Society is very cruel!* [...] *People look at me like I’m some kind of thing, or like I’m from another world!* (P4)

However, some survivors were able to reinterpret their self-image by assigning new meaning to their experience with the burn injury.

*I was born again!* [...] *It’s a new beginning for my life!* [...] *When I grow old and have grandchildren, it will be a life story I went through!* (P13)

### Financial challenges and impact on the role of provider

The inability to return to work created significant financial difficulties, especially for those who were the primary providers for their households. Being out of the workforce and facing unexpected medical expenses increased both economic and emotional insecurity.

*I was the one paying for everything-food, water, electricity, internet, and my children’s school!* [...] *I had a boyfriend who used to help me, but when I got sick, he abandoned me financially.* (P12)

In addition to the loss of income, survivors encountered bureaucratic obstacles in accessing social security benefits and reported a lack of support from their employers.

*They* [the company] *only started helping me after a photo of mine went viral on social media, but even then, it was very little. They brought some financial help to buy medicine* [while I was in the hospital], *and that was it!* (P13)

### Difficulties accessing treatment and maintaining continuity of care

Continuity of care after hospital discharge was another significant challenge. The lack of specialized services near the survivors’ homes, combined with the need to travel to reference centers, compromised adequate medical follow-up.

*I went to the physical therapist over a month ago to get home care arranged, and nothing yet* [...] *The services here aren’t reliable; you can’t really count on them.* (P3)

The precariousness of local services led some participants to rely on informal contacts, seeking guidance from professionals with whom they had built a bond during hospitalization.

*The doctors there* [...] *couldn’t tell that my wound was already infected!* [...] *based on what they had explained to me at* [the burn reference hospital], [...] *I knew it wasn’t normal!* [...] I *messaged a nurse I had become friends with* [at the burn reference hospital], *showed her a picture* [of the wound], *and* [...] *she showed it to the doctor, and he said, “You need to come here because I think that’s infected!” It was a struggle to get an ambulance to bring me because I couldn’t sit up!”* (P8)

### Overcoming challenges and receiving support in rebuilding life

Despite the difficulties, survivors reported various strategies to cope with the challenges imposed by the burn injury. Family support emerged as a central factor, providing emotional support and practical assistance.

*Family is very important!* [...] *I’m being very well cared for by my mother, children, and siblings* [...] *That’s made all the difference in my recovery.* (P2)

The presence of spouses, mothers, children, and friends was essential to the post-discharge adaptation process by minimizing feelings of loneliness and helplessness.

### Faith and spirituality in rebuilding life

Regardless of religious affiliation, faith was identified as a central element in coping with the new reality. Many survivors cited their belief in God as a source of strength and resilience. For some, spirituality was directly associated with emotional strengthening, helping them accept the changes imposed by the burn injury and build a new sense of purpose in life.

*God is giving me a chance to fight, to try to live the way it has to be from now on! The only one who can help is God.* (P5)

*God gave me strength* [...] *I know it was God who got me out of that hospital!* [...] *I was at risk of dying, and I know God gave me my life back! God gave me strength, and I overcame it!* (P7)

## DISCUSSION

The analysis of this study highlights that the hospital-to-home transition experienced by survivors of severe burn injuries is a challenging process, marked by physical, emotional, social, and financial difficulties. Although returning home represents the relief of leaving the hospital environment, it also triggers feelings of insecurity and anxiety in the face of the new demands of care and adaptation to the reality imposed by the burn injury.

One of the most significant challenges was persistent, difficult-to-manage pain, exacerbated by the lack of specialized support after discharge^(13)^. The absence of home-based assistance forced many survivors to seek relief on their own through either self-medication or informal support from family members and acquaintances. These findings confirm that burn-related pain is not merely a physical experience but also a multidimensional phenomenon associated with emotional suffering, altered self image, and social difficulties. Studies indicate that chronic pain in burn survivors is often related to social rejection and challenges in reintegrating into daily life, particularly in the workplace and social interactions^(14)^. The trauma of the burn injury, combined with prolonged hospitalization and uncertainty about recovery, places survivors in a state of extreme vulnerability, affecting their quality of life and emotional well-being.

The COVID-19 pandemic added a layer of complexity to the hospital-to-home transition. The restriction of visits during hospitalization limited family support and, consequently, increased survivors’ emotional dependence on the nursing team. This scenario may have contributed to an even more challenging post-discharge adaptation, as the absence of in-person contact with family members deprived survivors of one of their main emotional support mechanisms.

Some survivors mentioned the continuity of the bond with the multidisciplinary team after discharge as a positive factor in the adaptation process, providing reassurance and support for clarifying questions related to treatment. However, this experience was inconsistent across all survivors, indicating that maintaining this bond could be better structured to ensure systematic follow-up during the post-hospital period^(15)^. Continuous multidisciplinary support has been described in the literature as essential for minimizing complications and reducing the psychological impact of the transition to home^(16)^. In this regard, the presence of a designated professional capable of providing emotional support and case-specific guidance may promote a smoother adaptation and reduce the risk of physical and emotional complications.

Difficulties in social and professional reintegration were highlighted as central aspects of the survivors’ experiences. Physical limitations and changes in self-image had a direct impact on self-esteem and social interactions, leading many survivors to avoid public spaces and social interactions out of fear of rejection and judgment. Additionally, uncertainty about the ability to resume professional roles was a recurrent concern, especially among those who had been the primary providers for their households. The inability to return to work and resulting financial hardships generated additional stress, forcing survivors to rely on external support to secure basic needs. These findings reinforce the need for continuous multidisciplinary support through rehabilitation programs that address not only functional recovery but also psychological support and strategies for social and professional reintegration^(17)^.

Another critical aspect identified was the difficulty accessing treatment and maintaining continuity of care after discharge^(18)^. The precariousness of the public health network, the need to travel to specialized reference centers, and bureaucratic barriers made it difficult for survivors to receive appropriate medical follow-up, compromising their recovery. Many survivors turned to personal networks for support, revealing the fragility of outpatient care for this population. Additionally, delays in scheduling appointments and the shortage of specialized professionals in the participants’ regions further increased their vulnerability, highlighting gaps in the healthcare system that directly impact the quality of care provided^(19)^.

Faith and spirituality emerged as important strategies for coping with the challenges faced after the burn injury. For many survivors, belief in God and spiritual faith were sources of strength and resilience that helped them accept the changes imposed by the trauma and rebuild a new sense of purpose in life. The literature highlights that spirituality can serve as an effective coping mechanism, particularly in situations of physical and emotional suffering, thus promoting well-being and facilitating adaptation to adverse conditions^(20,21)^. In this context, recognizing the relevance of spirituality in the recovery process may contribute to providing more humanized care that aligns with the patients’ subjective needs^(22)^.

The findings of this study emphasize the need for public policies and institutional measures to ensure structured support for survivors of severe burn injuries. Among the priority actions is expediting access to social security benefits for workers who are on leave due to burn-related sequelae in order to reduce the financial impact of temporary or permanent disability. Improving communication between companies and injured workers is also essential, ensuring adequate support during recovery and preventing dismissal or work-related disadvantages^(23,24)^.

Expanding financial assistance to cover medical expenses not covered by the public healthcare system (SUS) is another key point to prevent survivors from relying solely on donations. In parallel, expanding the specialized Primary Care network for burn care would provide more accessible outpatient support and reduce the burden on reference hospitals. Finally, strengthening the coordination between healthcare services and social assistance programs would ensure more continuous and effective follow up for survivors facing physical and financial barriers to rehabilitation.

Implementing these strategies may help minimize financial impacts, ensure continuity of treatment, and facilitate survivors’ rehabilitation and social reintegration^(25)^. The findings of this study show that the hospital-to-home transition must be addressed comprehensively through multidisciplinary interventions that consider physical, emotional, and social aspects. The vulnerability of these survivors is influenced by multiple factors, such as the structure of the home environment and access to healthcare services, which can compromise the continuity of care. Therefore, discharge planning should include a personalized care plan tailored to the individual needs of each survivor, promoting adequate support and facilitating comprehensive recovery.

### Study limitations

This study presents some limitations that should be considered when interpreting the results. Data collection occurred during the COVID-19 pandemic, when sanitary restrictions significantly affected hospital care and the interaction between survivors and their families. The loneliness experienced by survivors during hospitalization and their emotional dependence on the nursing team were mentioned by some participants, which may have influenced their perceptions of the hospital to home transition. This context differs from periods without social distancing, in which the presence of family members in the hospital and the support of personal networks could mitigate some of the difficulties reported.

Another limitation concerns the sample profile. The study was conducted with survivors who received care at a reference hospital; therefore, this characteristic may limit the applicability of the findings to contexts with different healthcare structures, particularly in locations with limited access to specialized services.

Additionally, the analysis was based on survivors’ accounts and, therefore, reflects individual perceptions of the hospital-to-home transition process. Although the qualitative approach allows for a deep understanding of these experiences, it was not possible to quantify the frequency or intensity of the challenges reported, which may limit the generalization of the results to other populations of survivors of severe burn injuries.

Despite these limitations, the findings offer valuable contributions to understanding the needs and challenges faced by survivors in returning to daily life, highlighting aspects that can support improvements in multidisciplinary care during the post-discharge period.

### Contributions to the field of nursing

The results of this study allow healthcare professionals, especially nurses, to reflect on the importance of implementing a personalized care plan for the discharge of survivors of severe burn injuries. This planning should address not only the physical repercussions, such as pain and functional limitations, but also the emotional and social aspects involved in the process of adapting to life at home.

In addition, the study highlights shortcomings in referral and counter-referral processes, which compromise continuity of care and hinder positive rehabilitation outcomes for survivors. These weaknesses require ongoing attention from the coordinators of Healthcare Networks to promote improvements and ensure effective follow-up during the post-discharge period, strengthening home care services and preventing complications.

## CONCLUSION

This study investigated the perceptions of survivors of severe burn injuries regarding the hospital-to-home transition and revealed that this process is marked by physical, emotional, social, and financial challenges. The main difficulties faced by the participants included physical sequelae, such as persistent pain, scarring, and changes in body image, along with emotional distress associated with adapting to the new reality.

Social and professional reintegration also emerged as significant challenges, with survivors reporting social exclusion, absence from work activities, and decreased income. Difficulty accepting their new body image was a central issue in this process, affecting self-esteem and the resumption of social interactions. On the other hand, family support emerged as an essential factor in coping with these difficulties, providing emotional support and facilitating adaptation to the new life context.

Based on these findings, there is a clear need for actions that promote comprehensive and continuous care, focusing on support for social and occupational reintegration, psychological follow-up, and the strengthening of support networks. Nursing plays a fundamental role in this process, which should include the early identification of physical and emotional stressors and the assessment of each survivor’s individual needs, going beyond standardized care protocols.

The results of this study reinforce the importance of discharge planning and structured post hospital follow-up to minimize the negative impacts of the transition and support the comprehensive recovery of survivors of severe burn injuries.

## Data Availability

The research data supporting the findings of this study are openly available in the SciELO Data repository at the following DOI: https://doi.org/10.48331/SCIELODATA.CEENKV

## References

[1] World Health Organization (WHO) (2023). Burns: key facts.

[2] Lopes CD, Ferreira GLI, Adorno J. (2021). Manual de queimaduras para estudantes.

[3] Kim KJ, Boo S, Oh H. (2021). Burn survivors’ experiences of the ongoing challenges after discharge in South Korea: a qualitative study. Adv Skin Wound Care.

[4] Quinn L, Ahmed T, Falk H, Altamirano AM, Muganza A, Nakarmi K (2023). Burn admissions across lowand middle-income countries: a repeated cross-sectional survey. J Burn Care Res.

[5] Martins VC, Sousa GL, Tavares TC, Oliveira JMO, Almeida IC, Parreira SLS. (2020). Estudo epidemiológico dos pacientes vítimas de queimaduras, tratados em um ambulatório do Hospital Municipal na cidade de Anápolis. Rev Ciênc Méd Biol.

[6] Schaefer TJ, Szymanski DK. (2024). In: StatPearls.

[7] Bayuo J, Wong FKY. (2021). Issues and concerns of family members of burn patients: a scoping review. Burns.

[8] Gheno J, Weis AH. (2021). Care transition in hospital discharge for adult patients: integrative literature review. Texto Contexto Enferm.

[9] Wagner CM, Butcher HK, Clarke MF. (2023). Nursing Interventions Classification (NIC).

[10] Acosta AM, Lima MADS, Marques GQ, Zucatti PB, Silveira CS, Oelke ND. (2022). Development of a measurement instrument to assess patient safe transition at hospital discharge. Rev Gaúcha Enferm.

[11] Buus N, Perron A. (2020). The quality of quality criteria: replicating the development of the Consolidated Criteria for Reporting Qualitative Research (COREQ). Int J Nurs Stud.

[12] Braun V, Clarke V. (2022). Conceptual and design thinking for thematic analysis. Qual Psychol.

[13] Jawad AM, Kadhum M, Evans J, Cubitt JJ, Martin N. (2024). Recovery of functional independence following major burn: a systematic review. Burns.

[14] Carvalho RRS, Caminha ECCC, Leite ACS. (2019). A dor da queimadura: percepções de enfermeiras. Rev Bras Queimaduras.

[15] Campbell JM, Kavanagh S, Kurmis R, Munn Z. (2017). Systematic reviews in burns care: poor quality and getting worse. J Burn Care Res.

[16] Mardani A, Azizi M, Noodeh FA, Alizadeh A, Maleki M, Vaismoradi M (2024). A concept analysis of transitional care for people with cancer. Nurs Open.

[17] Luz RM, Oliveira AR, Moreno TES, Barbosa DA, Silva IL, Simoneti RAO. (2021). Aspectos psicológicos de pacientes pós-queimaduras: uma revisão da literatura. Braz J Dev.

[18] Cavalli GCP, Mosquéra JM, Ramos LFAL, Alves AR, Hanna MD, Napoli AER. (2019). Relação entre a qualidade das prescrições médicas e a compreensão do paciente: uma revisão de literatura. Braz J Health Rev.

[19] Albuquerque IM, Mesquita VR, Silva RM, Freitas CA, Ribeiro MA, Vasconcelos AM, Pequeno AMC, Holanda MA, Barros MCS, Ribeiro MCA (2019). Pesquisa para o SUS Ceará: coletânea de artigos do PPSUS 5.

[20] Alves MES, Barakat SH, Oliveira MPS, Lima BDS, Oliveira TR, Ferreira REB (2022). Espiritualidade e religiosidade em pacientes hospitalizados com dor crônica. Res Soc Dev.

[21] Santos NRP, Castro MMC. (2019). Chronic pain: perception of elderly cancer patients in hospital and their coping strategies. Rev Psicol Divers Saúde.

[22] Rodrigues LA, Poiati ML, Nogueira MJ, Andrade MO, Brandini NL, Rezende RB. (2019). O profissional de saúde na unidade de tratamento de queimados: atenção e cuidado com os aspectos psicológicos dos pacientes. Rev Bras Queimaduras.

[23] Katsu A, Mackenzie L, Tyack Z, Mackey M. (2024). Understanding return-to-employment experiences after burns: Qualitative scoping review findings. Aust Occup Ther J.

[24] Van Bentum J, Nicholoson J, Bale N, Fadyl JK. (2021). Supporting people experiencing a burn injury to return to work or meaningful activity: qualitative systematic review and thematic synthesis. N Z J Physiother.

[25] Jhunjhunwala R, Jayaram A, Mita C, Davies J, Chu K. (2024). Community support for injured patients: a scoping review and narrative synthesis. PLoS One.

