# Supplementary material for: Experiences of severe burn survivors in the hospital-to-home transition
Source: Rev Bras Enferm. 2025 Oct 3;78(4):e20240493. doi: 10.1590/0034-7167-2024-0493 (PMC12494351; doi:10.1590/0034-7167-2024-0493)
Supplement: Translation

# Translation: Experiências de sobreviventes de queimaduras graves na transição do hospital para o domicílio

**DOI:** 10.1590/0034-7167-2024-0493pt

**Authors:** Karina da Silva Vale Yagi, Vanessa da Silva Carvalho Vila, Maysa Ferreira Martins Ribeiro, Virginia Visconde Brasil

**Affiliations:** I Pontifícia Universidade Católica de Goiás. Goiânia, Goiás, Brasil; II Universidade Federal de Goiás. Goiânia, Goiás, Brasil

**Keywords:** Queimaduras, Alta do Paciente, Continuidade da Assistência ao Paciente, Cuidados de Enfermagem, Cuidado Transicional.

## Abstract

**Objetivo:**

Compreender a experiência vivida por sobreviventes de queimaduras graves no processo de transição do hospital para o domicílio.

**Métodos:**

Estudo qualitativo realizado com 13 sobreviventes (oito mulheres e cinco homens) em um centro de referência em Goiânia, Brasil. A coleta de dados incluiu prontuários, contatos telefônicos para confirmar a participação e entrevistas, tanto remotas quanto presenciais. As entrevistas foram gravadas, transcritas e analisadas por meio de análise temática interpretativa em seis etapas.

**Resultados:**

Os principais desafios enfrentados foram as sequelas físicas (dor, cicatrizes e mudanças na imagem corporal) e o sofrimento emocional. Barreiras para o retorno às atividades cotidianas incluíram exclusão social, afastamento do trabalho, redução de renda e dificuldade em aceitar a nova imagem corporal, enquanto o apoio familiar foi essencial.

**Conclusões:**

A transição para a vida pós-hospitalar requer cuidados abrangentes e contínuos, com foco na reinserção social, laboral e suporte emocional.

## INTRODUÇÃO

As queimaduras representam lesões teciduais orgânicas causadas por uma variedade de agentes, incluindo elementos químicos, térmicos, físicos e biológicos, resultando em feridas traumáticas com implicações físicas, psicológicas e estéticas significativas^(1,2)^. Indivíduos que sobrevivem a queimaduras enfrentam desafios multifacetados, que vão além dos riscos decorrentes da perda da integridade tecidual e abrangem o manejo da dor intensa, o impacto emocional caracterizado por ansiedade e estresse, bem como as dificuldades na adaptação às transformações físicas e funcionais impostas pela lesão^(1-3)^.

Globalmente as queimaduras são reconhecidas como um sério problema de saúde pública, com aproximadamente 180 mil mortes registradas anualmente, ao passo que, no Brasil, cerca de 2.500 mortes anuais são atribuídas a essa causa^(1)^. A incidência dessas lesões é mais pronunciada em países de baixa e média renda, sendo influenciada por condições socioeconômicas desfavoráveis, como moradias precárias, falta de educação, habitações superlotadas e deficiências nas campanhas de educação em saúde e prevenção^(4)^.

As queimaduras não fatais frequentemente acarretam complicações de longo prazo, abrangendo limitações físicas, cicatrizes permanentes e traumas psicológicos, que comprometem a qualidade de vida e a autonomia dos indivíduos, prejudicando suas atividades diárias, interações sociais e bem-estar^(1,5)^. A gravidade das lesões é determinada pela extensão da área corporal afetada e pela profundidade da queimadura, com lesões graves implicando danos mais extensos aos tecidos e maior risco de mortalidade^(6)^.

Após a hospitalização, o papel da família torna-se crucial no processo de recuperação do paciente, fornecendo suporte emocional e auxiliando na adesão ao tratamento^(7)^. A transição do ambiente hospitalar para o domicílio requer abordagem multidisciplinar para identificar as necessidades individuais do paciente e de sua família^(8)^. No entanto, o processo de alta hospitalar muitas vezes é negligenciado, resultando em transições inadequadas que podem comprometer a recuperação do paciente e sua reintegração à comunidade^(9,10)^.

A compreensão das experiências vividas pelos sobreviventes de queimaduras pode fornecer insights valiosos para o desenvolvimento de intervenções e políticas que visem melhorar o suporte oferecido durante a transição para a vida pós-hospitalar, preenchendo uma lacuna significativa na literatura e na prática clínica.

## OBJETIVO

Compreender a experiência vivenciada por sobreviventes de queimaduras graves no processo de transição do hospital para o domicílio.

## MÉTODOS

### Aspectos éticos

Os comitês de ética em pesquisa da Pontifícia Universidade Católica de Goiás e o do Centro de Assistência aos Radioacidentados Leide das Neves Ferreira aprovaram o estudo, que atendeu aos princípios éticos estabelecidos na Resolução 466/2012 do Conselho Nacional de Saúde. O Termo de Consentimento Livre e Esclarecido foi obtido de todos os participantes do estudo, em formato impresso ou digital, de acordo com a preferência de cada participante. Os sobreviventes foram identificados pela letra P, seguida de algarismo arábico.

### Desenho, período e local do estudo

Trata-se de um estudo com abordagem qualitativa, relatado com base nas diretrizes do *Consolidated criteria for reporting qualitative research* (COREQ)^(11)^. A coleta de dados foi realizada entre janeiro e junho de 2022, em um Centro de Referência em Assistência à Queimados de Alta Complexidade localizado na região metropolitana de Goiânia, estado de Goiás, Brasil.

### Amostra, critérios de inclusão e exclusão

O estudo contou com a participação de sobreviventes de queimaduras graves que estavam em acompanhamento no centro de referência durante o período de coleta de dados. A seleção dos sobreviventes iniciou-se com a identificação de indivíduos que haviam recebido alta hospitalar entre 30 e 180 dias antes da coleta, garantindo que tivessem passado pelo processo de adaptação ao domicílio, essencial para a apreensão de suas experiências.

Os critérios de inclusão foram: idade igual ou superior a 18 anos; queimaduras graves e complexas, definidas como lesões com extensão superior a 20%, profundidade de 2º grau profundo ou maior, queimaduras em áreas nobres e/ou queimaduras elétricas; alta hospitalar entre 30 e 180 dias antes da coleta; e acompanhamento pela equipe de cirurgia plástica.

Foram excluídos pacientes acompanhados pela equipe de cirurgia plástica por outros motivos, indivíduos cuja alta hospitalar tinha ocorrido há mais de seis meses e aqueles que tinham abandonado o tratamento ambulatorial. A avaliação da gravidade das queimaduras foi realizada paralelamente à verificação dos critérios de inclusão, uma vez que o estudo adotou parâmetros específicos para essa classificação. Assim, os critérios de inclusão já contemplavam a definição de gravidade utilizada.

Na coleta de dados, foram identificados 150 potenciais participantes com base na lista de pacientes agendados para retorno e consulta com a equipe multiprofissional do centro de referência. Aplicando os critérios de inclusão, verificou-se que 50 desses indivíduos eram elegíveis para o estudo, os quais foram contatados. No entanto, registraram-se 37 perdas, decorrentes de números de telefone desatualizados ou inválidos e recusas em participar da pesquisa. Logo, a amostra final do estudo foi composta por 13 sobreviventes de queimaduras graves.

Dado que a seleção dos participantes ocorreu em função da disponibilidade de pacientes em acompanhamento no centro de referência e da acessibilidade para contato e participação, a amostra foi caracterizada como não probabilística por conveniência. Esse método de amostragem foi adotado considerando as especificidades do estudo, incluindo a necessidade de identificar sobreviventes de queimaduras graves dentro de um intervalo específico de alta hospitalar e em acompanhamento ambulatorial, além da viabilidade de recrutamento dentro do período estabelecido para a pesquisa.

### Coleta de dados

A coleta de dados foi realizada em duas etapas. Inicialmente, os participantes foram identificados e selecionados com base no histórico clínico registrado no prontuário eletrônico da instituição. Esse processo permitiu a análise dos critérios de gravidade das queimaduras e a aplicação dos critérios de inclusão e exclusão do estudo. A pesquisadora obteve autorização para acessar a lista de pacientes agendados para retorno médico com a equipe de cirurgia plástica. Com isso, ela analisou relatórios médicos, registros de enfermagem e anotações da equipe multiprofissional, desde o atendimento inicial até a alta hospitalar.

Após essa etapa inicial, foi organizada uma lista contendo informações básicas dos pacientes, como contatos telefônicos e um breve histórico da hospitalização. Durante os contatos telefônicos, verificou-se que a maioria dos números cadastrados não pertencia diretamente aos sobreviventes, e sim a familiares, amigos ou instituições de apoio. Por essa razão, foram necessárias de duas a três tentativas de ligação para estabelecer o primeiro contato direto com o participante. Durante as chamadas, a pesquisadora se identificou, explicou os objetivos do estudo e forneceu todas as informações necessárias para que os sobreviventes tomassem uma decisão informada sobre sua participação. Após a confirmação verbal de interesse, foi oferecida a opção de entrevista on-line ou presencial.

No total, foram realizadas duas entrevistas presenciais e 11 entrevistas on-line, norteadas por um roteiro de entrevista semiestruturada explorando aspectos sociodemográficos e experiências relacionadas à vivência da queimadura. Exemplos de perguntas incluíram: “Você enfrentou dificuldades ao retornar para seu domicílio?”, “Qual o sentimento que você tem hoje em relação ao fato de ter se queimado?” e “O que ajudou você a enfrentar esse processo?”. Todas as entrevistas foram gravadas e identificadas numericamente para preservar o anonimato dos participantes.

O encerramento da coleta de dados ocorreu mediante o processo analítico, interativo e interpretativo, considerando o princípio da suficiência teórica, que considera a qualidade e a profundidade das informações obtidas como fatores determinantes para o encerramento da coleta. A decisão de encerrar a coleta de dados baseou-se na coerência analítica, garantindo que os temas emergentes estavam suficientemente desenvolvidos; na diversidade e variedade, assegurando múltiplas perspectivas sobre a vivência pós-queimadura; na densidade conceitual, com temas consistentes e sustentados por descrições detalhadas; e em um critério pragmático, considerando a viabilidade temporal e a disponibilidade de participantes.

Essa abordagem permitiu um encerramento criterioso da coleta, de modo que a análise temática fosse conduzida de maneira rigorosa, alinhada aos princípios metodológicos da abordagem reflexiva. Dessa forma, salientamos que a decisão de encerrar a coleta de dados não se baseou exclusivamente na ausência de novos dados, mas, sim, na suficiência das informações para responder aos objetivos do estudo, proporcionando profundidade e coerência interpretativa.

### Análise dos dados

Inicialmente, realizou-se a transcrição das falas logo após cada entrevista, com o auxílio de uma das autoras do estudo, após treinamento, orientação e supervisão da pesquisadora principal. Em seguida, os dados foram analisados por meio da Análise Temática Reflexiva^(12)^, um método interpretativo que enfatiza a construção ativa dos significados pelo pesquisador, permitindo uma leitura aprofundada e teoricamente informada do fenômeno estudado. A abordagem adotada foi indutiva, ou seja, os temas emergiram diretamente dos dados, sem a imposição de categorias prévias, permitindo que as experiências dos sobreviventes fossem analisadas em sua complexidade e singularidade.

O processo analítico seguiu seis etapas interconectadas. Primeiramente, foi realizada a familiarização com os dados, com leitura cuidadosa das transcrições e anotações iniciais sobre padrões emergentes. Em seguida, procedeu-se à identificação dos códigos, por meio de codificação linha a linha para destacar unidades de significado relevantes. Posteriormente, os códigos foram agrupados em temas, organizando-os em padrões mais amplos de significado com base em suas inter-relações. Essa estrutura foi refinada na revisão dos temas, promovendo coerência interna e distinção entre eles. Após esse refinamento, realizou-se a definição e nomeação dos temas, consolidando seus significados centrais e articulando sua fundamentação teórica. Por fim, os temas foram descritos e interpretados de forma narrativa, considerando a profundidade e complexidade dos significados expressos pelos sobreviventes.

À medida que a leitura do material se aprofundava, os códigos foram identificados de forma processual em todas as entrevistas, utilizando uma abordagem iterativa e reflexiva. Essa etapa incluiu uma leitura crítica dos dados, discussões analíticas com a pesquisadora principal e uma revisão contínua dos códigos emergentes. A construção dos temas ocorreu mediante agrupamento e refinamento progressivo dos códigos, culminando na estruturação de macrotemas que sintetizam as vivências dos participantes.

A decisão sobre o encerramento da análise seguiu o princípio da suficiência teórica, com prioridade para a profundidade e coerência interpretativa dos temas, em vez da noção tradicional de saturação de dados. Essa abordagem enfatiza um processo analítico contínuo e reflexivo, no qual a qualidade da interpretação se sobrepõe à simples repetição de informações. Para garantir clareza metodológica, adotou-se um conjunto de critérios que orientaram a definição do ponto de encerramento da análise^(12)^.

O primeiro critério foi a densidade conceitual, considerando que os temas emergentes demonstraram consistência e profundidade, sustentados por descrições detalhadas dos participantes, sem necessidade de novos dados para ampliar a compreensão do fenômeno. Em seguida, a coerência interna foi avaliada, assegurando que os códigos e temas identificados mantivessem relações lógicas e bem definidas. Isso refletiu padrões significativos dentro do conjunto de dados.

Também foi levada em conta a variabilidade das experiências, para certificar-se de que diferentes perspectivas fossem contempladas e representadas na análise, sem a necessidade de um volume maior de entrevistas para reforçar padrões já estabelecidos. O critério de estabilidade analítica também foi considerado, verificando se a interpretação dos dados permanecia consistente ao longo das diferentes etapas da análise, sem novas variações conceituais emergindo.

Outro aspecto fundamental foi a discussão por pares, realizada com a pesquisadora principal e outros membros da equipe, o que permitiu um olhar crítico e colaborativo sobre os achados e fortaleceu a validade interpretativa da análise. Por fim, aspectos pragmáticos foram levados em consideração, tais como a viabilidade temporal da pesquisa e a disponibilidade dos participantes, para sustentar um equilíbrio entre aprofundamento teórico e factibilidade do estudo. Com base nesses critérios, a análise foi conduzida de forma criteriosa e interativa, que assegurasse fidelidade às experiências narradas pelos participantes e coerência interna dos temas identificados.

## RESULTADOS

O estudo incluiu 13 sobreviventes de queimaduras, sendo oito (61,5%) mulheres e cinco (38,5%) homens, com idades entre 22 e 42 anos (média de 34,2 anos). Seis (46,2%) eram casados ou viviam em união estável, cinco (38,5%) eram solteiros e dois (15,3%) divorciados. Nove (69,2%) se identificaram como evangélicos e quatro (30,8%) como católicos. Oito (61,5%) tinham entre um e três filhos. Nove (69,2%) concluíram o ensino médio. A maioria tinha renda entre um e dois salários mínimos. Quanto à ocupação, seis (46,2%) tinham vínculo empregatício formal, um (7,7%) estava afastado por invalidez temporária e cinco (38,5%) eram autônomos, sem renda fixa.

A análise dos dados revelou que a transição do hospital para o domicílio foi um processo desafiador para os sobreviventes de queimaduras graves, pois envolveu múltiplas dimensões que influenciaram sua adaptação à nova realidade. Seis macrotemas emergiram da análise indutiva, os quais representaram as principais experiências e desafios enfrentados pelos sobreviventes: Dificuldades no retorno ao cotidiano; Impacto na autoimagem e na vida social; Desafios financeiros e impacto no papel de provedor; Dificuldades no acesso ao tratamento e continuidade do cuidado; Superação e apoio na reconstrução da vida; e Fé e espiritualidade.

Esses macrotemas estruturam a apresentação dos resultados e permitem uma compreensão abrangente das experiências dos sobreviventes. Os achados são ilustrados por excertos das falas dos sobreviventes, que evidenciam as nuances e significados atribuídos a cada aspecto vivenciado no período pós-alta hospitalar.

### Dificuldades no retorno ao cotidiano

O retorno para casa foi descrito pelos sobreviventes como um momento de intensa insegurança e medo. A ausência da equipe hospitalar gerou um sentimento de desamparo, que tornou a adaptação mais desafiadora.

*É assustador!* [...] *eu ainda tô meio que assim* [...] *sem acreditar!* [...] *a gente passa e pensa* [...] *eu mesma que estou passando por isso?!* (P2)

*Voltar é muito sofrido, dolorido e tudo faz você sofrer.* (P5)

Além disso, a dor persistente foi um dos principais desafios relatados, muitas vezes incontrolável com os medicamentos prescritos. Alguns sobreviventes buscaram alívio por conta própria, sem acompanhamento profissional.

*Tudo o que você faz, sente uma dor* [...] *é muita dor, é uma dor que é inexplicável!* [...] *é muito sensível a pele* [...] *você sente uma fisgada, você nunca esquece. Quando tenta esquecer, a própria ferida te lembra que está ali!* (P9)

*O médico que me deu alta, não me deu nenhum remédio para dor, me passou dipirona!* [...] *eu fiquei uns três dias sentindo dor em casa assim que eu cheguei!* [...] *teve um dia aqui em casa que eu estava chorando muito, não estava aguentando! Pedi socorro para minha vizinha que é enfermeira do Corpo de Bombeiros!* [...] *ela conseguiu uma receita de tramadol.* (P1)

As limitações motoras e a necessidade de evitar a exposição solar também impactaram a autonomia dos sobreviventes, porque restringiram sua mobilidade e aumentaram a dependência de terceiros para atividades diárias.

*Estou muito limitado* [...] *eu não saio no sol* [...] *ainda não consigo jogar vôlei* [...] *não dou conta de correr* [...] *não consigo andar direito.* (P1)

### Impacto na autoimagem e na vida social

A aceitação da nova imagem corporal foi um dos maiores desafios enfrentados pelos sobreviventes. As cicatrizes e a perda de cabelo foram fontes de angústia e baixa autoestima, dificultando o reconhecimento da própria aparência.

*Acho que a gente demora um pouco para se adaptar a tudo isso!* [...] *Eu não aceito o que aconteceu!* [...] *Tenho uma rejeição de mim mesma, de me olhar no espelho* [...] *A autoestima que eu tinha antes, não tenho mais!* (P8)

*Tento não olhar no espelho, ficaram [cicatrizes] fundas e horríveis! Se eu olhar, eu fico triste!* [...] *Se for para fazer correção* [cirurgia plástica], *eu quero fazer, eu não tenho medo!* (P12)

A percepção do olhar alheio também foi um fator impactante. Muitos sobreviventes relataram desconforto e constrangimento em espaços públicos, sentindo-se observados e, por vezes, excluídos do convívio social.

*A sociedade é muito cruel!* [...] *As pessoas olham como se eu fosse uma coisa ou uma pessoa de outro mundo!* (P4)

No entanto, alguns sobreviventes conseguiram ressignificar sua autoimagem mediante a atribuição de um novo significado à experiência da queimadura.

*Eu nasci de novo!* [...] *É um recomeço da minha vida!* [...] *Quando eu ficar velho e tiver neto, vai ser uma história de vida que eu passei!* (P13)

### Desafios financeiros e impacto no papel de provedor

A impossibilidade de retornar ao trabalho gerou dificuldades financeiras significativas, especialmente para aqueles que eram os principais provedores do lar. O afastamento do mercado de trabalho e os gastos médicos inesperados aumentaram a insegurança econômica e emocional.

*Era eu* [quem fazia] *as despesas da casa com comida, água, energia, internet e escola dos meus filhos!* [...] *Eu tinha um namorado que me ajudava, mas quando fiquei doente, ele me abandonou financeiramente.* (P12)

Além da perda de renda, os sobreviventes enfrentaram entraves burocráticos para acessar benefícios previdenciários e relataram a falta de suporte por parte de seus empregadores.

*Eles* [empresa] *começaram a me dar assistência depois que uma foto minha foi divulgada nas redes sociais, mas foi pouca. Levaram* [ajuda financeira] *para comprar remédio* [quando estava no hospital*], e foi só isso!* (P13)

### Dificuldades no acesso ao tratamento e continuidade do cuidado

A continuidade do cuidado pós-alta foi outro desafio significativo. A falta de serviços especializados próximos às residências, somada à necessidade de deslocamento para centros de referência, comprometeu o acompanhamento médico adequado.

*Fui lá na fisioterapeuta para ela atender aqui em casa já tem mais de mês, e até hoje nada* [...] *Os serviços aqui não adianta não, a gente não pode contar muito com eles.* (P3)

A precariedade no atendimento local levou alguns participantes a recorrerem a contatos informais, buscando orientação com profissionais com os quais haviam criado vínculo durante a hospitalização.

*Os médicos lá* [...] *não conseguiram analisar que a minha ferida já estava infectada!* [...] *pelo que me explicaram no* [no hospital referência em queimaduras], [...] *eu sabia que não era normal!* [...] *mandei mensagem para a enfermeira* [no hospital referência em queimaduras] *que* [...] *eu tinha amizade, mostrei a foto* [da ferida] *e* [...] *ela mostrou para o médico e ele mandou “ precisa vir para cá porque eu acho que isso daí infectou”! Foi uma dificuldade para conseguir uma ambulância para poder me levar, não tinha como eu ir sentada!”* (P8)

### Superação e apoio na reconstrução da vida

Apesar das dificuldades, os sobreviventes relataram diferentes estratégias para enfrentar os desafios impostos pela queimadura. O apoio familiar emergiu como um fator central nesse processo, de maneira que proporcionou acolhimento emocional e suporte prático.

*A família é muito importante!* [...] *Tô sendo muito bem cuidada pela minha mãe, filhos e irmãos* [...] *Tá sendo o diferencial para uma boa melhora.* (P2)

A presença de cônjuges, mães, filhos e amigos mostrou-se essencial para a adaptação pós-alta, por minimizar sentimentos de solidão e impotência.

### A fé e espiritualidade na reconstrução da vida

Independentemente da crença religiosa, identificou-se que a fé foi um elemento central no enfrentamento da nova realidade. Muitos relataram a crença em Deus como fonte de força e resiliência. Para alguns, a espiritualidade esteve diretamente associada ao fortalecimento emocional: auxiliou na aceitação das mudanças impostas pela queimadura e na construção de um novo propósito de vida.

*Deus tá me dando uma oportunidade pra tentar lutar, tentar viver do jeito que tem que ser de agora em diante! O único que pode ajudar é Deus.* (P5)

*Deus que me deu força* [...] *Eu sei que foi Deus que me tirou daquele hospital!* [...] *Corri risco de vida, eu sei que Deus me devolveu a vida! Deus me deu força e eu superei!* (P7)

## DISCUSSÃO

A análise deste estudo evidencia que a transição hospitalar para o domicílio vivenciada por sobreviventes de queimaduras graves é um processo desafiador, permeado por dificuldades físicas, emocionais, sociais e financeiras. Embora o retorno para casa represente o alívio de deixar o ambiente hospitalar, ele também desperta sentimento de insegurança e ansiedade diante das novas demandas de cuidado e adaptação à realidade imposta pela queimadura.

Um dos desafios mais marcantes foi a dor persistente e de difícil manejo, agravada pela falta de suporte especializado após a alta^(13)^. A ausência de assistência domiciliar obrigou muitos sobreviventes a buscarem alívio por conta própria, seja por meio de automedicação ou pelo suporte informal de familiares e conhecidos. Esses achados confirmam que a dor decorrente de queimaduras não é apenas uma experiência física, mas também um fenômeno multidimensional, associado ao sofrimento emocional, à alteração da autoimagem e às dificuldades sociais. Estudos apontam que a dor crônica em sobreviventes de queimaduras está frequentemente relacionada à rejeição social e às dificuldades na reintegração à vida cotidiana, especialmente no âmbito do trabalho e do convívio social^(14)^. O trauma da queimadura, somado ao longo período de hospitalização e à incerteza sobre a recuperação, coloca os sobreviventes em um estado de extrema vulnerabilidade, impactando sua qualidade de vida e bem estar emocional.

A pandemia de covid-19 impôs uma camada adicional de complexidade à transição para o domicílio. A restrição de visitas durante a internação limitou o suporte familiar e, consequentemente, aumentou a dependência emocional dos sobreviventes em relação à equipe de enfermagem. Esse cenário pode ter contribuído para uma adaptação pós-alta ainda mais desafiadora, já que a ausência de contato presencial com familiares restringiu um dos principais mecanismos de apoio emocional.

A continuidade do vínculo com a equipe multiprofissional após a alta foi mencionada por alguns sobreviventes como um fator positivo no processo de adaptação, que proporcionou segurança e suporte para esclarecer dúvidas sobre o tratamento. No entanto, essa experiência não foi uniforme entre os sobreviventes, e isso indica que a manutenção desse vínculo poderia ser mais bem estruturada, com vistas a garantir um acompanhamento sistemático no período pós-hospitalar^(15)^. O suporte multiprofissional contínuo tem sido descrito na literatura como essencial para minimizar complicações e reduzir o impacto psicológico da transição para o lar^(16)^. Nesse sentido, a presença de um profissional de referência, capaz de fornecer suporte emocional e orientações específicas para cada caso, pode favorecer uma adaptação mais tranquila e reduzir o risco de agravos físicos e emocionais.

As dificuldades na reintegração social e profissional foram evidenciadas como aspectos centrais na experiência dos sobreviventes. As limitações físicas e as mudanças na autoimagem geraram impactos diretos na autoestima e no convívio social, o que levou muitos sobreviventes a evitarem interações e espaços públicos devido ao receio da rejeição e do julgamento alheio. Além disso, a insegurança quanto à capacidade de reassumir funções profissionais foi um fator de preocupação recorrente, especialmente para aqueles que eram os principais provedores do lar. A impossibilidade de retornar ao trabalho e as dificuldades financeiras geraram estresse adicional, exigindo a dependência de apoio externo para garantir a subsistência. Esses achados reforçam a necessidade de suporte multiprofissional contínuo, por meio de programas de reabilitação que contemplem tanto a recuperação funcional quanto o apoio psicológico e estratégias para a reinserção social e profissional^(17)^.

Outro aspecto crítico identificado foi a dificuldade no acesso ao tratamento e na continuidade do cuidado pós-alta^(18)^. A precariedade da rede de saúde pública, a necessidade de deslocamento para centros de referência e os entraves burocráticos dificultaram o acompanhamento médico, e isso comprometeu a recuperação dos sobreviventes. Muitos recorreram às redes pessoais para obter suporte, o que demonstrou a fragilidade da assistência ambulatorial para esse grupo de sobreviventes. Além do mais, a demora na liberação de consultas e a falta de profissionais especializados nas regiões de residência dos participantes ampliaram sua vulnerabilidade, evidenciando lacunas no sistema de saúde que impactam diretamente a qualidade da assistência prestada^(19)^.

A fé e a espiritualidade emergiram como estratégias importantes no enfrentamento dos desafios pós-queimadura. Para muitos sobreviventes, a crença em Deus e a fé foram fontes de força e resiliência que auxiliaram na aceitação das mudanças impostas pelo trauma e na reconstrução de um novo significado para suas vidas. A literatura destaca que a espiritualidade pode atuar como um mecanismo de enfrentamento eficaz, especialmente em situações de sofrimento físico e emocional; assim, ela favorece o bem-estar e a adaptação a condições adversas^(20,21)^. Nesse contexto, reconhecer a relevância da espiritualidade no processo de recuperação pode contribuir para um cuidado mais humanizado e alinhado às necessidades subjetivas dos pacientes^(22)^.

Os achados deste estudo ressaltam a necessidade de políticas públicas e medidas institucionais que garantam suporte estruturado para sobreviventes de queimaduras graves. Entre as ações prioritárias, destaca-se a agilização do acesso a benefícios previdenciários para trabalhadores afastados por sequelas de queimadura, a fim de reduzir o impacto financeiro da incapacidade temporária ou permanente. Também é essencial aprimorar a comunicação entre empresas e trabalhadores acidentados, com garantia de suporte adequado durante a recuperação e prevenção de demissões ou prejuízos laborais^(23,24)^.

A ampliação da assistência financeira para cobrir despesas médicas não contempladas pelo SUS é outro ponto fundamental para evitar que os sobreviventes dependam exclusivamente de doações. Paralelamente, a expansão da rede de Atenção Primária especializada em queimaduras permitiria um suporte ambulatorial mais acessível e reduziria a sobrecarga dos hospitais de referência. Por fim, fortalecer a articulação entre os serviços de saúde e a assistência social garantiria um acompanhamento mais contínuo e efetivo para sobreviventes que enfrentam barreiras físicas e financeiras na reabilitação.

A implementação dessas estratégias pode minimizar os impactos financeiros, assegurar a continuidade do tratamento e facilitar a reabilitação e reinserção social dos sobreviventes^(25)^. Os achados deste estudo evidenciam que a transição hospitalar para o domicílio deve ser abordada de maneira integral, por meio de intervenções multiprofissionais que contemplem os aspectos físicos, emocionais e sociais. A vulnerabilidade desses sobreviventes é influenciada por múltiplos fatores, como a estrutura do ambiente doméstico e o acesso aos serviços de saúde, que podem comprometer a continuidade dos cuidados. Dessa forma, o planejamento da alta deve incluir um plano de cuidados personalizado, adaptado às necessidades individuais de cada sobrevivente, de modo que promova suporte adequado e favoreça a recuperação integral.

### Limitações do estudo

Este estudo apresenta algumas limitações que devem ser consideradas na interpretação dos resultados. A coleta de dados ocorreu durante a pandemia de covid-19, quando as restrições sanitárias impactaram significativamente a assistência hospitalar e a interação entre sobreviventes e familiares. A solidão vivenciada pelos sobreviventes durante a internação e a dependência da equipe de enfermagem para suporte emocional foram mencionadas por alguns participantes, o que pode ter influenciado suas percepções sobre a transição para o domicílio. Esse contexto difere de períodos sem distanciamento social, nos quais a presença de familiares no hospital e o suporte da rede de apoio podem reduzir parte das dificuldades relatadas.

Outra limitação refere-se ao perfil amostral. O estudo foi conduzido com sobreviventes atendidos em um hospital de referência; logo, essa característica pode restringir a aplicabilidade dos achados a contextos com diferentes estruturas de assistência, especialmente em locais com menor acesso a serviços especializados.

Além disso, a análise dos dados se baseou nos relatos dos sobreviventes, portanto reflete percepções individuais sobre o processo de transição hospitalar. Embora a abordagem qualitativa permita uma compreensão aprofundada dessas experiências, não foi possível quantificar a frequência ou a intensidade dos desafios relatados, o que pode limitar a extrapolação dos resultados para outras populações de sobreviventes de queimaduras graves.

Apesar dessas limitações, os achados oferecem contribuições relevantes para a compreensão das necessidades e desafios enfrentados pelos sobreviventes no retorno à vida cotidiana, destacando aspectos que podem subsidiar a melhoria da assistência multiprofissional no período pós-alta.

### Contribuições para a área da enfermagem

Os resultados deste estudo permitem aos profissionais da saúde, especialmente aos enfermeiros, refletir sobre a importância da implementação de um plano de cuidados personalizado para a alta de sobreviventes de queimaduras graves. Esse planejamento deve considerar não apenas as repercussões físicas, como dor e limitações funcionais, mas também os aspectos emocionais e sociais envolvidos no processo de adaptação ao domicílio.

Além disso, o estudo evidencia falhas nos processos de referência e contrarreferência, que comprometem a continuidade do cuidado e dificultam a obtenção de desfechos positivos na reabilitação dos sobreviventes. Essas fragilidades exigem atenção contínua dos coordenadores das Redes de Atenção à Saúde, a fim de promover melhorias e garantir um acompanhamento eficaz no período pós-alta, fortalecendo a assistência domiciliar e prevenindo complicações.

## CONCLUSÕES

Este estudo investigou a percepção de sobreviventes de queimaduras graves sobre a transição do hospital para o domicílio e evidenciou que esse processo é marcado por desafios físicos, emocionais, sociais e financeiros. As principais dificuldades enfrentadas pelos participantes incluíram sequelas físicas, como dor persistente, cicatrizes e alterações na imagem corporal, além do sofrimento emocional associado à adaptação à nova realidade.

A reinserção social e profissional também se mostrou um desafio significativo, com sobreviventes relatando exclusão social, afastamento das atividades laborais e redução da renda. A dificuldade em aceitar a nova imagem corporal foi um aspecto central nesse processo, que interferiu na autoestima e na retomada das interações sociais. Por outro lado, o apoio familiar emergiu como um fator essencial para o enfrentamento das dificuldades, o qual ofereceu suporte emocional e facilitou a adaptação ao novo contexto de vida.

Diante desses achados, ressalta-se a necessidade de ações que promovam cuidados abrangentes e contínuos, com foco no suporte à reinserção social e laboral, acompanhamento psicológico e fortalecimento das redes de apoio. O papel da enfermagem é fundamental nesse processo: deve incluir a identificação precoce de fatores de estresse físico e emocional, bem como a avaliação das necessidades individuais dos sobreviventes, indo além dos protocolos padronizados de cuidado.

Os resultados desta pesquisa reforçam a importância do planejamento da alta e do acompanhamento pós-hospitalar estruturado, a fim de minimizar os impactos negativos da transição e favorecer a recuperação integral dos sobreviventes de queimaduras graves.

## Data Availability

Os dados de pesquisa que fundamentam este estudo estão disponíveis no repositório Scielo Data, acessível por meio do seguinte DOI: https://doi.org/10.48331/SCIELODATA.CEENKV
